# Language and terminology to describe transgender communities; Perspectives from people with lived experience

**DOI:** 10.1080/26895269.2024.2415684

**Published:** 2024-10-15

**Authors:** Sav Zwickl, Foster Skewis, Tomi Ruggles, Joel Murray, Teddy Cook, Ada S. Cheung

**Affiliations:** a Trans Health Research Group, Department of Medicine (Austin Health), The University of Melbourne, Melbourne, Australia; b Neophile, Sydney, Australia; c The Kirby Institute, University of New South Wales, Sydney, Australia; d Gender Clinic, Department of Endocrinology, Austin Health, Melbourne, Australia

**Keywords:** Gender diverse persons, language, non-binary persons, terminology, transgender persons

## Abstract

**Background:**

There is no consensus over the meaning of “transgender,” “trans and gender diverse,” and other terminology used to describe people with a gender different from the gender presumed or recorded for them at birth. We aimed to survey transgender (trans) individuals to understand lived experience perspectives on terminology.

**Methods:**

An online cross-sectional survey of trans people currently living in Australia and aged ≥16 was conducted from June 2021 to April 2022. Demographic data and terminology preferences were ascertained through fixed-option and free-text questions.

**Results:**

Of 1023 respondents with a median age of 30 years (IQR 24, 41), 37.1% were trans women, 34.9% were trans men, 27.6% were non-binary, and 0.4% had a culturally specific identity. There was no consensus regarding community-level terminology, with “trans/transgender and gender diverse” the most preferred term (21.9%), followed by “trans/transgender” (19.3%), and “gender diverse” (15.1%). The term “trans/transgender” was more preferred by trans women (27.9%), trans men (19.0%), respondents who had a history of or wanted gender-affirming hormone therapy (GAHT; 20.7%), and gender-affirming surgery (GAS; 20.9%), compared to non-binary respondents (8.2%), and respondents who did not want GAHT (4.9%) or GAS (13.9%). Three themes emerged from free-text responses regarding term preference: (1) Inclusivity, (2) Trans as a binary and medicalized experience, and (3) Societal understanding, accessibility, and destigmatization.

**Conclusion:**

From Australian trans communities’ perspectives, the term “trans/transgender and gender diverse” was the most preferred community-level terminology to collectively describe people with a gender different from the gender presumed or recorded for them at birth. Further perspectives are needed from other cohorts internationally.

## Introduction

People with a gender different from the gender presumed or recorded for them at birth are a highly diverse population in terms of gender identity, gender expression, and desires for medical gender affirmation, including gender-affirming hormone therapy (GAHT) and gender-affirming surgery (GAS). This population is also diverse in their understandings and preferences for the terminology that is used to describe their personal gender identity and gender experiences, and their broader communities.

On an individual level, it is well-established best practice that the terminology used to refer to an individual should be guided by that individual (Coleman et al., 2022). However, when it comes to community-level terminology—terminology used in reference to all people with a gender different from the gender presumed or recorded for them at birth—there is no established consensus.

Community-level terminology used by governments, community organizations, support groups, researchers, and healthcare professionals varies greatly. For example, on an international level, the World Professional Association for Transgender Health (WPATH) uses “transgender and gender diverse” in the Standards of Care 8 (SoC8) (Coleman et al., 2022), while the European Professional Association for Transgender Health (EPATH) and the Professional Association for Transgender Health Aotearoa (PATHA) use “transgender” (EPATH, 2024; PATHA, 2024). Within Australia, the terminology also varies. For example, the Australian Professional Association for Trans Health (AusPATH) uses “trans—binary and non-binary” (AusPATH, 2024), the Australian Bureau of Statistics (ABS) uses “trans and gender diverse” (ABS, 2021), and community organization Transcend use “transgender, gender diverse and non-binary” (Transcend, 2024).

Language is integral to ensuring that intended populations are sufficiently targeted, represented, and included across a range of settings. Therefore, a lack of consensus around best-practice terminology has implications for belonging and sense of community, access to healthcare, policy, and research, all of which may, in turn, impact health and well-being outcomes.

We aimed to survey individuals with a gender different from the gender presumed or recorded for them at birth, to understand community perspectives on community-level language and terminology. Given the strong binary and medicalized notions of “trans/transgender” (Vipond, 2015), we hypothesized that respondents with a binary gender identity and those who have had or want medical gender affirmation would be more likely to view that term as most inclusive. By comparison, those respondents with a non-binary gender identity, and those not interested in medical gender affirmation, would be more likely to prefer other terminology.

## Methods

Participants in the online cross-sectional survey were drawn from the TRANSform study, an ongoing longitudinal investigation into trans health in Australia, initiated in May 2020. Eligible TRANSform participants—individuals aged 16 and above residing in Australia, whose gender is different from the gender presumed or recorded for them at birth—were recruited through non-probability snowball sampling *via* social media and various trans community support networks and organizations in Australia.

TRANSform participants complete an enrollment survey that involves providing basic demographic details and an email address. Subsequently, they receive email invitations to engage in two or three TRANSform sub-studies annually. The survey discussed in this article replaced the previous TRANSform enrollment survey on June 11, 2021, and was extended to existing participants through personalized email links. Starting from June 11, 2021, all newly recruited TRANSform participants were required to complete this enrollment survey upon joining the longitudinal study. Data for this article was collected between June 11, 2021, and April 21, 2022. While written informed consent was not obtained, participants were informed in the survey preamble that completion implied consent.

The survey design was a collaborative effort involving researchers from Australian trans and gender diverse communities, a research advisory group comprising trans and gender diverse individuals, and specialized clinicians in trans healthcare. Data collection and management utilized REDCap electronic tools hosted at The University of Melbourne. Ethical approval was granted by the Austin Health Human Research Ethics Committee (Reference Number HREC/57155/Austin-2019), ACON Research Ethics Review Committee (Reference Number 2020/03), and the Thorne Harbor Health Community Research Endorsement Panel (Reference Number THH/CREP 20-006).

Demographic data collected included date of birth, Aboriginal or Torres Strait Islander heritage, country of birth, sex assigned at birth, innate variations of sex characteristics, and state/territory of residence. To ascertain personal gender identity, respondents were asked to select the term(s) that best described their current gender from 41 fixed-response options, including an “other” option that allowed for a free-text response. For data analysis purposes, respondents also categorized themselves into one of four fixed-response options: man/trans man, woman/trans woman, non-binary or gender diverse (including agender) (herein referred to as non-binary), and a culturally specific identity not covered by the available options (herein referred to as culturally specific identity). Questions regarding history and desire for gender-affirming hormone therapy (GAHT) and gender-affirming surgery (GAS) were presented with fixed-option responses, encompassing a range of treatments and procedures used for gender affirmation.

To explore preferences for community-level terminology, respondents were asked to provide one fixed-option response to the question “What resonates most with you as an inclusive term to describe our entire community (i.e. an umbrella term)?” Twenty-two common variations of community-level terminology were presented in alphabetical order, including an “other” option that allowed for a free-text response. To further understand preference responses, respondents were asked to provide a free-text response to “Can you tell us more about why you use this option?”

Respondent demographic data and rates of preference for terminology are reported as frequency (percentage) for categorical variables, and median (interquartile range), as appropriate for not normally distributed data. Free-text responses to the three most preferred terms were collated and imported into NVivo qualitative data analysis software version 14 (QSR International Pty Ltd., Doncaster, Australia) for thematic analysis. One of the researchers (FS) coded participant responses one-by-one, and two other members of the research team (SZ, TR) reviewed the coded dataset, and discrepancies and queries with the coding of discrete data points were discussed and resolved. Codes were developed during analysis and were not previously established. The research team collectively allocated the codes to broader themes.

## Results

### Respondent demographics

A total of 1067 survey responses were received. After removing duplicate and incomplete responses, 1023 valid responses remained. The median age of the respondents was 30 years (IQR 24, 41), 37.1% (*n* = 380) were trans women, 34.9% (*n* = 357) were trans men, 27.6% (*n* = 282) were non-binary, and 0.4% (*n* = 4) had a culturally specific identity. More detailed personal gender identity data is available in Supplementary Appendix A. A majority of respondents were born in Australia (82.6%, *n* = 845), which is higher than the Australian general population (ABS, 2024), and Aboriginal or Torres Strait Islander heritage was reported by 3.9% (*n* = 40), which is representative of the general Australian population (ABS, 2022). Additional demographic data of the survey respondents is shown in Table 1.

**Table 1.** Participant characteristics.

Variable	*N*	%
Age (years) (*n* = 1010)		
16–25	321	31.8
26–35	345	34.2
36–45	146	14.5
46–55	107	10.6
56–65	59	5.8
66+	32	3.2
Location (*n* = 1023)		
Australian Capital Territory	40	3.9
New South Wales	247	24.1
Northern Territory	9	0.9
Queensland	173	16.9
South Australia	58	5.7
Tasmania	26	2.5
Victoria	380	37.1
Western Australia	90	8.8
Aboriginal or Torres Strait Islander (*n* = 1023)		
Aboriginal	39	3.8
Aboriginal and Torres Strait Islander	1	0.1
Non-indigenous	957	93.5
Unsure	16	1.6
Prefer not to say	10	1.0
Country of birth (*n* = 1023)		
Australia	845	82.6
United Kingdom	67	6.5
Aotearoa New Zealand	27	2.6
United States of America	19	1.9
Malaysia	9	0.9
Singapore	7	0.7
Hong Kong	6	0.6
China	5	0.5
Other	38	3.7
Sex/gender assigned at birth (*n* = 1023)		
Female	576	56.3
Male	429	41.9
No birth certificate	1	0.1
Prefer not to say	17	1.7
Variations of sex characteristics (Intersex) (*n* = 1023)		
Yes	26	2.5
Unsure	115	11.2
No	877	85.7
Prefer not to say	5	0.5
Limited gender category (*n* = 1023)		
Trans women	380	37.1
Trans men	357	34.9
Non-binary or gender diverse (including agender)	282	27.6
A culturally specific identity not captured by other options	4	0.4
History/interest in GAHT (*n* = 1020)		
Currently using GAHT	851	83.4
Taking steps to start GAHT	54	5.3
Thinking about if GAHT is right for them	70	6.9
No interest in GAHT	41	4.0
Unsure	3	0.3
Prefer not to say	1	0.1
History/interest in GAS (*n* = 1020)		
Has had one or more GAS	401	39.3
Taking step to have GAS	275	27.0
Thinking about if GAS is right for them	252	24.7
No interest in GAS	81	7.9
Unsure	7	0.7
Prefer not to say	4	0.4

GAHT: gender affirming hormone therapy; GAS: gender affirming surgery.

### Terminology preferences

In total, “trans/transgender and gender diverse” was the most preferred community-level terminology (21.9%, *n* = 224), followed by “trans/transgender” (19.3%, *n* = 197) and “gender diverse” (15.1%, *n* = 154). When stratified by gender, trans women were the most likely to prefer “trans/transgender” (27.9%, *n* = 106), compared to trans men (19.0%, *n* = 68) and non-binary respondents (8.2%, *n* = 23). Inversely, non-binary respondents were most likely to prefer “trans/transgender and gender diverse” (29.1%, *n* = 82), compared to trans men (20.2%, *n* = 72) and trans women (18.2%, *n* = 69). Three of the four respondents with a culturally specific identity preferred the term “gender diverse” (Table 2).

**Table 2.** Preference for community-level terminology by gender identity.

Community terminology	Trans men *N* (%) (*N* = 357)	Trans women *N* (%) (*N* = 380)	Non-binary *N* (%) (*N* = 282)	Culturally specific identity *N* (%) (*N* = 4)	Total sample *N* (%) (*N* = 1023)
Gender diverse	56 (15.7)	57 (15.0)	38 (13.5)	3 (75.0)	154 (15.1)
Gender expansive	2 (0.6)	3 (0.8)	3 (1.1)	0 (0.0)	8 (0.8)
Gender non-compliant	0 (0.0)	1 (0.3)	2 (0.7)	0 (0.0)	3 (0.3)
Gender non-conforming	5 (1.4)	2 (0.5)	5 (1.8)	0 (0.0)	12 (1.2)
Genderqueer	6 (1.7)	7 (1.8)	8 (2.8)	0 (0.0)	21 (2.1)
Queer	27 (7.6)	17 (4.5)	17 (6.0)	0 (0.0)	61 (6.0)
Trans/transgender	68 (19.0)	106 (27.9)	23 (8.2)	0 (0.0)	197 (19.3)
Trans/transgender and gender expansive	3 (0.8)	2 (0.5)	2 (0.7)	0 (0.0)	7 (0.7)
Trans/transgender and gender diverse	72 (20.2)	69 (18.2)	82 (29.1)	1 (25.0)	224 (21.9)
Trans/transgender and non-binary	8 (2.2)	10 (2.6)	13 (4.6)	0 (0.0)	31 (3.0)
Trans/transgender, gender diverse, and non-binary	26 (7.3)	18 (4.7)	25 (8.9)	0 (0.0)	69 (6.7)
Trans/transgender, gender diverse, and nonconforming	15 (4.2)	12 (3.2)	12 (4.3)	0 (0.0)	39 (3.8)
Trans*	13 (3.6)	21 (5.5)	5 (1.8)	0 (0.0)	39 (3.8)
Trans* and gender expansive	1 (0.3)	1 (0.3)	2 (0.7)	0 (0.0)	4 (0.4)
Trans* and gender diverse	26 (7.3)	8 (2.1)	20 (7.1)	0 (0.0)	54 (5.3)
Trans* and non-binary	2 (0.6)	6 (1.6)	5 (1.8)	0 (0.0)	13 (1.3)
Trans*, gender diverse, and nonbinary	6 (1.7)	7 (1.8)	8 (2.8)	0 (0.0)	21 (2.1)
Trans*, gender diverse, and nonconforming	5 (1.4)	4 (1.1)	3 (1.1)	0 (0.0)	12 (1.2)
Transsexual	2 (0.6)	5 (1.3)	0 (0.0)	0 (0.0)	7 (0.7)
Other	2 (0.6)	7 (1.8)	3 (1.1)	0 (0.0)	12 (1.2)
Unsure	9 (2.5)	12 (3.2)	5 (1.8)	0 (0.0)	26 (2.5)
Prefer not to say	3 (0.8)	5 (1.3)	1 (0.4)	0 (0.0)	9 (0.9)

When stratified by medical gender affirmation status, respondents who had a history of or wanted GAHT were four times more likely to prefer “trans/transgender” (20.7%, *n* = 187), compared to respondents who did not want GAHT (4.9%, *n* = 2) (Table 3). Different rates of preference for “trans/transgender” were also observed in respondents with a history of or desire for GAS (20.9%, *n* = 141), compared to respondents who did not want GAS (13.9%, *n* = 11). “Trans/transgender and gender diverse” was the most preferred term by respondents who did not want GAHT (29.3%, *n* = 12) or GAS (21.5%, *n* = 17).

**Table 3.** Preference for community-level terminology by hormone and surgery status.

Community terminology	No GAHT *N* (%) (*N* = 41)	Have/want GAHT *N* (%) (*N* = 905)	No GAS *N* (%) (*N* = 79)	Have/want GAS *N* (%) (*N* = 676)	Full sample *N* (%) (*N* = 1023)
Gender diverse	6 (14.6)	138 (15.2)	13 (16.5)	107 (15.8)	154 (15.1)
Gender expansive	1 (2.4)	6 (0.7)	1 (1.3)	3 (0.4)	8 (0.8)
Gender non-compliant	0 (0.0)	3 (0.3)	1 (1.3)	1 (0.1)	3 (0.3)
Gender non-conforming	1 (2.4)	11 (1.2)	1 (1.3)	8 (1.2)	12 (1.2)
Genderqueer	1 (2.4)	17 (1.9)	3 (3.8)	9 (1.3)	21 (2.1)
Queer	0 (0.0)	54 (6.0)	5 (6.3)	38 (5.6)	61 (6.0)
Trans/transgender	2 (4.9)	187 (20.7)	11 (13.9)	141 (20.9)	197 (19.3)
Trans/transgender and gender expansive	0 (0.0)	6 (0.7)	1 (1.3)	5 (0.7)	7 (0.7)
Trans/transgender and gender diverse	12 (29.3)	200 (22.1)	17 (21.5)	153 (22.6)	224 (21.9)
Trans/transgender and non-binary	1 (2.4)	26 (2.9)	2 (2.5)	19 (2.8)	31 (3.0)
Trans/transgender, gender diverse, and non-binary	6 (14.6)	55 (6.1)	10 (12.7)	44 (6.5)	69 (6.7)
Trans/transgender, gender diverse, and nonconforming	1 (2.4)	36 (4.0)	3 (3.8)	24 (3.6)	39 (3.8)
Trans*	0 (0.0)	36 (4.0)	1 (1.3)	25 (3.7)	39 (3.8)
Trans* and gender expansive	1 (2.4)	3 (0.3)	0 (0.0)	4 (0.6)	4 (0.4)
Trans* and gender diverse	1 (2.4)	47 (5.2)	3 (3.8)	32 (4.7)	54 (5.3)
Trans* and non-binary	2 (4.9)	11 (1.2)	1 (1.3)	10 (1.5)	13 (1.3)
Trans*, gender diverse, and nonbinary	2 (4.9)	15 (1.7)	2 (2.5)	13 (1.9)	21 (2.1)
Trans*, gender diverse, and nonconforming	0 (0.0)	7 (0.8)	0 (0.0)	7 (1.0)	12 (1.2)
Transsexual	0 (0.0)	6 (0.7)	0 (0.0)	5 (0.7)	7 (0.7)
Other	1 (2.4)	11 (1.2)	1 (1.3)	9 (1.3)	12 (1.2)
Unsure	2 (4.9)	22 (2.4)	3 (3.8)	13 (1.9)	26 (2.5)
Prefer not to say	1 (2.4)	8 (0.9)	0 (0.0)	6 (0.9)	9 (0.9)

GAHT: gender affirming hormone therapy; GAS: gender affirming surgery; Respondents who indicated that they were “thinking about,” “unsure,” or “prefer not to say” to questions about gender-affirming hormones and surgery were excluded from the GAHT and GAS columns but included in the total sample figures.

### Themes underlying terminology preference

Qualitative analysis of the free-text responses for the three terms with the highest rates of preference (“trans/transgender and gender diverse,” “trans/transgender,” and “gender diverse”), resulted in the emergence of three overarching themes; (1) Inclusivity, (2) Trans as a binary and medicalized experience, and (3) Societal understanding, accessibility, and destigmatization. Themes and sub-themes are outlined in Supplementary Appendix B.

#### Theme 1: Inclusivity

Inclusivity was the most prevalent theme across the free-text responses. Among respondents who preferred “trans/transgender,” it was often understood as an umbrella term, encompassing many different gender identities and experiences. Some respondents emphasized the importance of “trans/transgender” as a singular term in relation to community solidarity, policy, and advocacy:

‘Trans’ is an inclusive umbrella term to describe broadly that your gender doesn’t align with your sex assigned at birth. All trans people, whether they have a binary gender or are non-binary have shared experiences, and uniting under this one term is important for our collective solidarity. – Non-binary person, 20’s

I feel like it’s more empowering for us to all unify under ‘trans’ rather than suggest ‘trans’ is a different category to ‘gender diverse and nonbinary’. – Non-binary person, 20’s

Further, some respondents described how deviation from ‘trans/transgender’ as a standalone term, can be harmful, potentially contributing to the loss of nuance and division within communities:

‘Trans’ is an umbrella term… The idea that there needs to be more explaining or caveats often stems from a belief that there is a need to differentiate between ‘binary’ and ‘nonbinary’ in a harmful way, as there are many steps between that don’t fit neatly. – Trans man, 20’s

Phrases like ‘trans and nonbinary’ can make some nonbinary [people] feel excluded because they already consider themselves transgender – nonbinary is just a more nuanced term for their identity. – Trans woman, 30’s

I feel like splitting it into ‘trans’ and ‘gender diverse’, while more inclusive on the outside, seems like it is dividing people up by whether they are ‘trans enough’ to be identified as trans. – Trans man, 30’s

Although this study did not make the distinction between “trans” and “transgender,” some respondents defined these two terms differently, which then had implications regarding inclusion. Most notably, “trans” may be more inclusive as an umbrella term rather than “transgender,” as some transsexual people do not consider themselves transgender:

I understand ‘trans’ as an inclusive umbrella term and ‘transgender’ as a specific label, but because trans is also shorthand for transgender, it can cause ambiguity. – Trans man, 20’s

‘Trans’ [is] a broad umbrella term that can include many kinds of identifications and experiences including transsexuals. – Non-binary person, 30’s

However, many respondents who preferred “trans/transgender and gender diverse” and “gender diverse,” recognized that there are some individuals who do not identify with the term “trans/transgender”:

Not all gender diverse people identify as transgender, so [‘trans/transgender and gender diverse’] seems the most inclusive, and ‘gender diverse’ includes identities that expand past the gender binary. – Trans woman, 40’s

Additionally, cultural inclusion was also a consideration of many respondents who conveyed recognition of “trans/transgender” as a Western term and notion, and stated the need for community-level terminology to be inclusive of non-Western genders:

I think that ‘trans’ or ‘transgender’ are good words, but they don’t always cover people of other cultural backgrounds who have maybe never had an AGAB [assigned gender at birth] or those who were raised without gender, which ‘gender diverse’ does. – Trans man, 30’s

‘Gender diverse’ includes cultural identities outside of trans, acknowledges gender diversity as being culturally significant beyond the label of trans – a Western term that functions as a colonial identity. – Non-binary person, 20’s

Some respondents who preferred “gender diverse” as a community-level term, described it as the least specific, and therefore, the most inclusive, without potential for hierarchies:

‘Gender Diverse’ captures the diversity of the community, without emphasizing specific labels which people may not identify with. – Trans man, 30’s

If we need an umbrella, ‘gender diverse’ is short, inclusive, and nonspecific/non-hierarchical (not putting trans before anyone else’s identity). – Trans man, 20’s

#### Theme 2: Trans as a binary and medicalized experience

Contrary to some respondents who understood “trans/transgender” as an umbrella term encompassing all people with a gender different from the gender presumed or recorded for them at birth, other respondents referenced the binary and medicalized notions associated with the term. Many of these respondents, therefore preferred “trans/transgender and gender diverse” or “gender diverse,” as more inclusive of non-binary people and people who do not pursue medical gender affirmation:

‘Trans’ covers people that are more likely to be on the binary spectrum and ‘gender diverse’ is more likely to cover the non-binary side of the spectrum. – Non-binary person, 30’s

‘Trans’… can sometimes infer medical transition. – Non-binary person, teen.

If only ‘trans’ or ‘transgender’ is used, it is not always understood to include non-binary. – Trans woman, 60’s

#### Theme 3: Societal understanding, accessibility, and destigmatization

Along with consideration of communities’ understandings of terminology, broader societal understanding also emerged as a common theme. Some respondents reported that they preferred a term based on its familiarity with the broader cisgender population who may not understand more nuanced language:

It’s a struggle to find a term that makes sense to us but also is okay for people outside our community. I don’t always want to pander to them but at this stage of our existence and precarious legal, health and social status I am conscious of using terms that people who have no idea of our community might understand. – Trans woman, 40’s

[‘Trans/transgender’] is the most common and I think it’s well understood, a lot of the others are also a bit of a mouthful. – Non-binary person, 20’s

[‘Trans/transgender’ is] simple to say and accessible for autistic people and people with learning disabilities, using speech devices, etc. – Trans woman, 30’s

Furthermore, some respondents stressed the need for community terms that hold positive connotations and are destigmatizing:

‘Gender diverse’ sounds inclusive and positive – almost as if the world can embrace and celebrate such diversity. I feel like sadly there is a lot of stigma and negative connotations connected with a lot of the other terms. – Non-binary person, 20’s

## Discussion

This cross-sectional survey of 1023 individuals living in Australia, aged over 16 and with a gender different from the gender presumed or recorded for them at birth, demonstrates that there is no clear consensus regarding inclusive and encompassing community-level terminology. The terms with the greatest preference by this sample were “trans/transgender and gender diverse” (21.9%), “trans/transgender” (19.3%), and “gender diverse” (15.1%). Terms that are frequently used in research, treatment guidelines, and policy, such as “trans/transgender, gender diverse, and non-binary” and “trans/transgender and non-binary” were only preferred by 6.7 and 3% of respondents, respectively.

### Sociocultural drivers of lack of consensus

The lack of consensus on community-level terminology demonstrated in this study, is, in large part, due to contention around the meaning of the term “trans/transgender.” The diverse perspectives conveyed in this research are likely reflective of historical and ongoing sociocultural drivers that have resulted in shifts in the definition of the term over the last 25 years.

“Transgender” first gained popularity amongst communities in the 1990s. As a community-coined term, “transgender” acted to counter the binary, medicalized, and pathologized notion of “transsexual” which was prevalent in diagnosis and treatment guidelines, including earlier editions of the Diagnostic and Statistical Manual of Mental Disorders (DSM), the International Classification of Disease (ICD) and WPATH Standards of Care. Initially, transgender was a broader term that encompassed a range of experiences, including individuals who do not want to pursue medical gender affirmation and those individuals who have a gender that does not exclusively align with the woman-man gender binary (Bornstein, 1994; Feinberg, 2006 [1992]).

This broad, encompassing understanding of “transgender” was evident in many of the perspectives captured in Theme 1: Inclusion, whereby some respondents saw “trans/transgender” as an inclusive and encompassing umbrella term for all people with a gender different to the gender presumed or recorded for them at birth. As several respondent quotes illustrated, concern was expressed that deviation from a standalone term could potentially lead to division within communities.

However, over the past two decades, the meaning of “transgender” has narrowed, to increasing use as exclusively in reference to people with a binary gender and those who pursue medical gender affirmation. This is particularly apparent in mainstream media and medical discourse (Vipond, 2015). The shift to a more binary and medicalized notion of “transgender” has had implications in terms of its understanding as an umbrella term and the inclusion of non-binary people and those who do not pursue medical affirmation. For example, in the Non-binary people’s experiences in the UK report (Valentine, 2016), 65% of non-binary respondents considered themselves transgender, while 15% did not, and 20% were unsure. In the 2015 US Transgender Survey, 82% of non-binary respondents expressed that they were “very comfortable,” “somewhat comfortable,” or “neutral” with the word “transgender” being used to describe them (James et al., 2016).

This narrowed understanding of “trans/transgender” was also highlighted by Theme 2: Trans as a binary and medicalized experience, with respondents referencing the notion of “trans/transgender” as reserved for people with a binary gender identity (trans women and trans men) and people who pursue medical gender affirmation. Such respondents, therefore, understood terms, such as “trans/transgender and gender diverse” and “gender diverse” as more inclusive, particularly in consideration of individuals who do not consider themselves “trans/transgender,” and those who do not pursue medical affirmation, have a non-binary gender, and/or have a non-Western culturally specific identity or experience.

Additionally, notions of “trans/transgender” as binary and medicalized were also apparent in the patterns of terminology preference based on gender identity and medical gender affirmation status. Consistent with our hypotheses, respondents with a binary gender and those who reported a history of or desire for GAHT or GAS were most likely to prefer “trans/transgender,” while respondents with a non-binary gender and those who did not want GAHT or GAS were most likely to prefer “trans/transgender and gender diverse.”

Notably, commonly used terms, such as “trans/transgender, gender diverse and non-binary” and “trans/transgender and non-binary” were not commonly preferred by survey respondents. The inclusion of both “trans/transgender” and “non-binary” in a community-level term can infer that non-binary people are not trans/transgender and contribute to the notion of “trans/transgender” as a term reserved for binary gender identification.

Further, as captured by Theme 3: Societal Understanding, accessibility, and destigmatization, respondents also contended with language accessibility and the desire for positive connotations and destigmatization of the terminology used for their communities. However, it is binary gender, normative gender expression, and medicalized notions of the trans experience that are best understood by broader society. Therefore, there is a risk that in trying to make community-level terminology more accessible, diversity and nuances of communities will be excluded. This exclusion is most likely to impact non-binary people, Indigenous people, people of color, and those of other intersectional experiences.

### Considerations and recommendations for community-level terminology

This study demonstrates that different understandings of the term “trans/transgender” have an ongoing impact on preference for community-level terminology, and more broadly, on cohesion and inclusivity of communities. As highlighted in this study, considerations around community-level terminology need to recognize that colonization, racism, and Western ethnocentric understandings of gender have an enduring impact on Indigenous Peoples and people of color. Trans health has been largely US and Western-European centric (Coleman et al., 2022), and the application of Western terms to non-Western contexts can act to privilege the Western gender binary and contribute to the erasure of non-Western identities (Billard & Nesfield, 2020). WPATH’s SoC8 recommends that culturally relevant language should be used across different global settings (Coleman et al., 2022). It is integral that community-level terminology is directed by communities, and that Indigenous and non-Western Peoples perspectives are meaningfully represented and integrated into research, treatment guidelines, and policy.

While this study did not differentiate between “trans” and “transgender,” emerging literature and lived-experience perspectives indicate that the two may be considered as distinct terms, rather than “trans” being considered as simply a shortened version of “transgender.” The implications of this differentiation are particularly relevant to the inclusion of transsexual people under a community-level umbrella term. While “transsexual” is sometimes referred to as an outdated term (GLAAD, 2024), it is important to recognize that there are some people who are transsexual and not transgender. The use of “transgender” in community-level terminology may potentially exclude some transsexual people, whereas “trans” may be a more inclusive umbrella term, encompassing both transgender and transsexual people.

The lack of consensus and the diverse range of understandings of community-level terminology highlights the need for clear definitions to be used in research, treatment guidelines, policy, and other settings that pertain to people with a gender different from the gender presumed and recorded for them at birth. The SoC8 has a dedicated chapter to terminology, which outlines the decision-making process around the terminology used in the guideline, recognition of limitations in that process, and the need for the use of culturally relevant language across different global settings (Coleman et al., 2022).

The SoC8 states:

We use the phrase transgender and gender diverse (TGD) to be as broad and comprehensive as possible in describing members of the many varied communities globally of people with gender identities or expressions that differ from the gender socially attributed to the sex assigned to them at birth. This includes people who have culturally specific and/or language-specific experiences, identities or expressions, and/or that are not based on or encompassed by Western conceptualizations of gender, or the language used to describe it.

As is recognized in the SoC8, terminology continues to change, and it is important to be open to emerging understandings and uses of language. In this vein, our recommendation in terms of terminology and definition differs from that of the SoC8 in several ways. Firstly, due to the growing recognition of “trans” and “transgender” as two distinct terms, with implications in terms of inclusion, we use the term “trans” rather than “transgender.” Secondly, as the use of acronyms and initialisms can diminish the identities and experiences of marginalized peoples (Australian Government Style Manual: Aboriginal and Torres Strait Islander Peoples, 2024; McGuire, 2023; Riggs et al., 2024), we refrain from using acronyms or initialisms for this population. Thirdly, we recommend consideration of the accessibility and appropriateness of language across different settings and applications, and the need to ensure understanding by a broad range of people. This may include explicit inclusion of particular gender terms, including culturally specific terms particular to a population.

For example, within the Australian context we currently use the following statement in our research materials and outputs, with explicit mention of Aboriginal Sistergirls and Brotherboys:

Trans and gender diverse (hereafter referred to as trans) people have a gender that differs from the gender presumed or recorded for them at birth. This includes trans men, trans women, non-binary people, and people who have culturally specific and/or language-specific experiences, such as Sistergirls and Brotherboys.

While both “trans and gender diverse” and “gender diverse” were in the three most preferred community-level terms in this community sample and we currently use “gender diverse” in our research material, it should be noted that “gender diverse” and “gender diversity” are also used as population terms (i.e. as terms inclusive of all or varied gender identities and expressions, including cisgender people). This may cause confusion, particularly within policy and law reform settings. There is, therefore, a need for further consideration of the use of this term, in consultation with communities.

### Limitations

This cross-sectional study with survey respondents who were recruited online using a non-probability snowball sampling method has inherent limitations. The sample had few Aboriginal and Torres Strait Islander respondents and respondents with a culturally specific gender, and none of the twenty-two variations of community-level terminology provided in the survey explicitly included culturally specific gender identities, such as Sistergirls and Brotherboys. These limitations highlight the need for further research into the experiences and perspectives of Indigenous Peoples, racially marginalized peoples, and peoples of non-Western cultural backgrounds.

Consistent with previous research (Callander et al., 2021; Veale et al., 2019), the sample was also relatively young [median age 30 years (IQR 24,41)] and there were significantly more non-binary respondents presumed and recorded female at birth compared to male at birth. This may limit the generalizability of the findings. Sociodemographic characteristics, such as education, employment, and regional or remote location, may impact terminology preference, though were not ascertained. Extrapolation of findings to populations in other countries should also be approached with caution, given differences in sociocultural context.

This study did not differentiate between “trans” and “transgender” nor explore communities’ perspectives on the use of acronyms and initialisms (e.g. TGD for trans and gender diverse). This data was collected during 2021 and 2022, and given that terminology is rapidly changing, communities’ perspectives may have since shifted with the broader use of a more expansive definition of “trans,” including some community organizations adopting terms, such as “trans people of all genders” and “trans people (binary and non-binary).” Although certain contexts may necessitate the use of community-level terminology, it cannot be assumed that all people with a gender different from the gender presumed or recorded for them at birth consider themselves as one community. Further research is needed to explore communities’ perspectives of these community-level terms, acronyms, and initialism, to ensure implementation of best-practice language by government, community organizations, support groups, researchers, and healthcare professionals.

## Conclusions

In this large cross-sectional survey of individuals living in Australia, aged over 16 and with a gender different from the gender presumed or recorded for them at birth, there was no clear consensus on community-level terminology. “Trans/transgender and gender diverse” and “trans/transgender” were the most preferred community-level terms, while other terms that are frequently used in research, policy, and by government and community organizations, such as “trans/transgender, gender diverse and non-binary” and “trans/transgender and non-binary” were not commonly preferred. Understanding lived experience perspectives on community-level terminology is integral to ensuring that intended communities are targeted, represented, and included across healthcare, health policy, community, and research settings. The use of community-level language that does not reflect the understandings of targeted communities, may exacerbate existing disparities in representation and health inequities within these communities. Given that sociocultural factors underlie understanding and use of terminology, further research is needed with gender diverse cohorts internationally, with a strong focus on the perspectives of Indigenous Peoples and racially marginalized peoples.

## Supplementary Material

Supplementary Material.docx

## Data Availability

Deidentified participant data are available upon reasonable request from the corresponding author *via* email (sav.zwickl@unimelb.edu.au), provided that the related research is deemed to be of benefit to the trans and gender diverse communities and has undergone Austin Health Human Research Ethics Committee approval in the form of an amendment.
