# Supplementary material for: Language and terminology to describe transgender communities; Perspectives from people with lived experience
Source: Int J Transgend Health. 2024 Oct 15;27(2):708–18. doi: 10.1080/26895269.2024.2415684 (PMC13015020; doi:10.1080/26895269.2024.2415684)
Supplement: Supplementary Material.docx [file WIJT_A_2415684_SM6739.docx]

**Appendix A.**

**Personal gender identity**

| Personal gender terms | Trans Men  N (%)  (N = 357) | Trans Women  N (%)  (N = 380) | Non-Binary  N (%)  (N = 282) | Cultural Identity  N (%)  (N = 4) | Total Sample  N (%)  (N = 1023) |
| --- | --- | --- | --- | --- | --- |
| Agender | 4 (1.1) | 5 (1.3) | 58 (20.6) | 0 (0.0) | 71 (6.9) |
| Androgynous | 4 (1.1) | 3 (0.8) | 23 (8.2) | 0 (0.0) | 31 (3.0) |
| Ay'lonit | 0 (0.0) | 0 (0.0) | 0 (0.0) | 1 (25.0) | 1 (0.1) |
| Bakla | 0 (0.0) | 0 (0.0) | 0 (0.0) | 1 (25.0) | 1 (0.1) |
| Bigender | 2 (0.6) | 2 (0.5) | 4 (1.4) | 0 (0.0) | 8 (0.8) |
| Brotherboy | 8 (2.2) | 0 (0.0) | 0 (0.0) | 1 (25.0) | 9 (0.9) |
| Demi boy/man | 8 (2.2) | 0 (0.0) | 13 (4.6) | 0 (0.0) | 21 (2.1) |
| Demi girl/woman | 0 (0.0) | 17 (4.5) | 11 (3.9) | 0 (0.0) | 28 (2.7) |
| Enby | 7 (2.0) | 18 (4.7) | 69 (24.5) | 0 (0.0) | 96 (9.4) |
| Gender Diverse | 23 (6.4) | 21 (5.5) | 52 (18.4) | 2 (50.0) | 101 (9.9) |
| Gender expansive | 1 (0.3) | 3 (0.8) | 9 (3.2) | 1 (25.0) | 14 (1.4) |
| Gender non-conforming | 21 (5.9) | 10 (2.6) | 51 (18.1) | 0 (0.0) | 83 (8.1) |
| Genderfluid | 3 (0.8) | 9 (2.4) | 35 (12.4) | 1 (25.0) | 48 (4.7) |
| Genderqueer | 24 (6.7) | 15 (3.9) | 77 (27.3) | 2 (50.0) | 120 (11.7) |
| Man | 171 (47.8) | 0 (0.0) | 4 (1.4) | 2 (50.0) | 177 (17.3) |
| Non-binary | 43 (12.0) | 41 (10.8) | 239 (84.8) | 3 (75.0) | 333 (32.6) |
| Queer | 53 (14.8) | 35 (9.2) | 104 (36.9) | 2 (50.0) | 198 (19.4) |
| Sistergirl | 0 (0.0) | 6 (1.6) | 0 (0.0) | 0 (0.0) | 6 (0.6) |
| Third gender | 2 (0.6) | 1 (0.3) | 0 (0.0) | 1 (25.0) | 4 (0.4) |
| Trans | 142 (39.7) | 103 (27.1) | 126 (44.7) | 3 (75.0) | 380 (37.1) |
| Transgender | 147 (41.1) | 172 (45.3) | 102 (36.2) | 1 (25.0) | 426 (41.6) |
| Trans feminine | 1 (0.3) | 122 (32.1) | 25 (8.9) | 1 (25.0) | 149 (14.6) |
| Trans man | 249 (69.6) | 0 (0.0) | 12 (4.3) | 1 (25.0) | 262 (25.6) |
| Trans masculine | 132 (36.9) | 0 (0.0) | 95 (33.7) | 1 (25.0) | 230 (22.5) |
| Trans woman | 0 (0.0) | 239 (62.9) | 5 (1.8) | 0 (0.0) | 244 (23.9) |
| Transsexual | 23 (6.4) | 45 (11.8) | 3 (1.1) | 0 (0.0) | 72 (7.0) |
| Two spirit | 4 (1.1) | 5 (1.3) | 0 (0.0) | 0 (0.0) | 9 (0.9) |
| Whakawahine | 0 (0.0) | 1 (0.3) | 0 (0.0) | 0 (0.0) | 1 (0.1) |
| Woman | 0 (0.0) | 169 (44.5) | 4 (1.4) | 1 (25.0) | 174 (17.0) |
| X-gender | 0 (0.0) | 3 (0.8) | 3 (1.1) | 0 (0.0) | 6 (0.6) |
| Cannot classify self | 0 (0.0) | 1 (0.3) | 8 (2.8) | 0 (0.0) | 10 (1.0) |
| Unsure/Questioning | 3 (0.8) | 5 (1.3) | 5 (1.8) | 0 (0.0) | 14 (1.4) |
| Other culturally specific term | 0 (0.0) | 0 (0.0) | 0 (0.0) | 1 (25.0) | 1 (0.1) |
| Other* | 3 (0.8) | 5 (1.3) | 10 (3.5) | 0 (0.0) | 18 (1.8) |
| Prefer not to say | 0 (0.0) | 1 (0.3) | 0 (0.0) | 0 (0.0) | 1 (0.1) |

*Free-text ‘other’ responses included 3 spirited, butch, demigender, dykefag, femme-them, fluidflux, FTM, genderfaun, genderflux, genderfuck, girl, hybrid human, intergender, maverique, nymphgender, other, plant, postgender, and xenic. Fixed response options that were not endorsed by any respondents have been excluded from this table, including Akava'ine, Fa'afafine, Fakaleiti, Hijra, Mahu, Mahu Vahine, Muxe, and Waria.

**Appendix B.**

**Overview of the themes and sub-themes for free-text responses regarding community-level terminology preferences**

| Theme | Sub-themes | Theme summary |
| --- | --- | --- |
| 1.Inclusivity | 1.1 Trans as an umbrella term | Understandings of ‘*trans/transgender*’ as an umbrella term, conveying community solidarity, with deviation of this term potentially contributing to loss of nuance and community division. |
|  | 1.2 Inclusion of those who don’t call themselves trans | Understandings of ‘*trans/transgender and gender diverse*’ and ‘*gender diverse*’ as more inclusive of those individuals who do not identify with the term ‘trans.’ |
|  | 1.3 Cultural inclusion | Understandings of ‘*trans/transgender’* as a Western term and notion, and recognition of other terminology as more inclusive of non-Western genders. |
| 2.Trans as a binary and medicalized experience | 2.1 Trans as binary, gender normative, medical | Understandings of ‘*trans/transgender*’ as tied to binary, gender normative, and medicalized experiences, and therefore, potentially not inclusive of non-binary and non-medicalized experiences. |
| 3.Societal understanding, accessibility and destigmatization | 3.1 Accessibility of language | Considerations of within-communities and broader societal understandings of terminology. |
|  | 3.2 Positive connotations and destigmatization | Considerations of positive connotations and destigmatizing potential of terminology. |
